# Virus based Full Colour Pixels using a Microheater

**DOI:** 10.1038/srep13757

**Published:** 2015-09-03

**Authors:** Won-Geun Kim, Kyujung Kim, Sung-Hun Ha, Hyerin Song, Hyun-Woo Yu, Chuntae Kim, Jong-Man Kim, Jin-Woo Oh

**Affiliations:** 1 Department of Nano Fusion Technology, Pusan National University; 2 Department of Cogno-Mechatronics Engineering, Pusan National University, Busan 609-735, Republic of Korea; 3 Department of Nanoenergy Engineering, Pusan National University, Busan 609-735, Republic of Korea

## Abstract

Mimicking natural structures has been received considerable attentions, and there have been a few practical advances. Tremendous efforts based on a self-assembly technique have been contributed to the development of the novel photonic structures which are mimicking nature’s inventions. We emulate the photonic structures from an origin of colour generation of mammalian skins and avian skin/feathers using M13 phage. The structures can be generated a full range of RGB colours that can be sensitively switched by temperature and substrate materials. Consequently, we developed an M13 phage-based temperature-dependent actively controllable colour pixels platform on a microheater chip. Given the simplicity of the fabrication process, the low voltage requirements and cycling stability, the virus colour pixels enable us to substitute for conventional colour pixels for the development of various implantable, wearable and flexible devices in future.

Since the beginning of life on Earth, evolution, by means of natural selection, has produced numerous nanomaterials, of which only the best are chosen and propagated through subsequent generations in the form of proteins and genes^1^,^2^. Proteins and genes mutually orchestrate spatial and temporal control over the synthesis of organic and inorganic nanomaterials, commonly resulting in hierarchical structures with specific functions^3^,^4^,^5^. Further modification of the structures to enable their manipulation has been a critical component of evolutionary pressure by means of natural selection. For example, a brittle star collects more light in the abyss using an array of inorganic calcite lenses^6^; *Morpho* butterflies communicate with one another over long distances using flickering colours produced by periodic layers of cuticles^7^, and many birds, such as turkeys and ostriches, respond to a situation with a display triggered by hierarchical protein structures in their skin or feathers^8^,^9^. Coherent scattering of white light by periodic three-dimensional structures in the skin of mammalian and avian produces constructive interference of light^9^,^10^. Owing to the fundamental properties of photonic crystals, which can be tuned by external stimulation^11^,^12^,^13^,^14^, mammalian and avian has been used directly as a colourimetric sensor for chemical analysis^15^.

A variety of inorganic and organic nanomaterial-based photonic crystals have been investigated to mimic biological functions through artificial fabrication processes^16^,^17^,^18^,^19^,^20^,^21^,^22^. The biomimetic approaches using top-down lithographic techniques for light manipulation have demonstrated great promise for various applications^19^,^23^. However, the artificial fabrication processes present considerable challenges for engineers since the fabrication of complex three-dimensional structures is difficult, expensive and time-consuming using top-down approaches. Thus, a bottom-up technique based on the self-assembly of primary building blocks has been investigated as an alternative to the top-down lithographic technique^24^,^25^,^26^. Fabrication by self-assembly using synthesised materials also requires considerable engineering efforts to produce highly stable primary building blocks and uniform size distributions. In addition, high-yield production of the uniform structures is difficult to achieve using the synthesised materials^27^,^28^. Attempts have been made to compensate for these disadvantages via novel approaches using well-defined natural biomaterials^29^,^30^,^31^. Here, we describe a temperature-dependent colour-generation platform based on optimisation of self-assembled structures of filamentous bacteriophage (M13 phage) (Fig. 1). We also explore the feasibility of a new class of display devices using highly stable and uniform structures.

The M13 phage is composed of single-stranded DNA encapsulated by 2,700 copies of the major coat protein pVIII. The length and width of M13 phage are typically ~880 nm and ~6.6 nm, respectively. The high aspect ratio allows for liquid crystallinity of the M13 phage. M13 phages can also easily amplify themselves in a highly regular manner through infection of bacterial host cells. Owing to these unique characteristics, researchers have explored the use of M13 phage as a primary building block in self-assembly processes^32^,^33^,^34^,^35^,^36^. Virus-based photonic crystal structure have no need for a filter layer to generate red, green, and blue (RGB) colours, so the fabrication is easier and more efficient than that of conventional colour pixels that are based mainly on silicon. Our group has introduced the M13 phage film based biosensor in the previous research^15^. The M13 phage film was successfully applied to a passive device for varied molecule adsorption. Interestingly, we found that the M13 phage film on the specific substrate can also display desired colours, which can be used for a more efficient and potential active device.

Utilizing M13 bacteriophage, we fabricated natural colour-generating self-assembled matrices (Fig. 2). By controlling the competing interfacial interaction at the meniscus, we can make M13 phage display various different colours, including white and black. The morphology of the M13 phage film can be easily modified by adjusting the chemical and kinetic conditions, resulting in expression of the desired colour. Atomic force microscopy (AFM) measurements and fast Fourier transform (FFT) analyses of the AFM images for the different matrices confirm that the generated colours are strongly dependent on the morphology of the M13 phage matrix (Fig. 2b,c). With a low pulling speed, the bundle becomes thicker and the bundle-to-bundle distance increases (Supplementary Fig. 1). In addition, constructive interference of the light scattered from the M13 phase colour film with a thick bundle and a long bundle-to-bundle distance occurs at a long wavelength. By contrast, constructive light interference of the film with thin bundle and short bundle-to-bundle distance occurs mainly at a short wavelength. Therefore, specific structures even generate whiteness and blackness (Supplementary Figs. S2 and S3). To obtain a better understanding of the wavelength shift measured in our experiments, we performed a simulation using a rigorous coupled wave analysis, which successfully explained the near- and far-field simulation (Fig. 2d). The M13 virus was modelled as a random array of phages with their coordinates, average sizes, and concentrations matching the AFM image in Fig. 2b. The reflectance measurements and simulation data showed good correlation with the FFT analysis (Fig. 2d). Moreover, the calculated near-field distribution of the samples confirmed the wavelength-shift for maximum reflectance (Supplementary Fig. 4). Randomly localised fields in the near-field distributions clearly showed intensity differences at different wavelengths. Thus, the numerical results suggest that both the M13 virus bundle concentration and the thickness tend to affect the excitation intensity significantly.

A back-substrate, such as the pigment layer of the birds, plays an important role in the colour-generation system of nature^37^. We mimicked the pigment layer by using different coating materials: a Si wafer coated with a 5-nm-thick Pt adhesion layer and a 100 nm-thick gold (Au) film (Au wafer), a Si wafer with a 2-nm-thick native SiO_2_ layer (Si wafer), a Si wafer with a thermally grown 300-nm-thick SiO_2_ layer (SiO_2_ wafer), and a bare glass slide. M13 phage colour films were prepared under the same self-assembly conditions on each substrate. FFT analysis of the AFM images confirmed the equivalent spatial order of each of three samples (Supplementary Fig. S5). Those samples present different reflectance values (Fig. 2f), due to the different optical properties of the substrate materials. The Au wafer surface absorbs primarily at short wavelengths and reflects the long wavelengths present in white light. By contrast, the SiO_2_ wafer surface absorbs primarily at long wavelengths and reflects in a short wavelength band (Supplementary Fig. S6). The transparency of the glass slide makes the virus colour film on it appear nearly transparent. Compared with the reference substrate, the virus colour film on the Au wafer shows a red-shifted reflectance, whereas the virus colour film on the SiO_2_ substrate shows a blue-shifted reflectance. Thus, we can enhance the targeted colour by choosing an appropriate substrate material with a different reflectance.

To demonstrate the performance of the virus colour film as a colour pixel, we integrated the film with a temperature-controllable microheater chip, which was fabricated by a standard metal lift-off process (Fig. 3a). In detail, a 1.4-μm-thick photoresist (PR; AZ5214, Clariant) mould with a negative sidewall profile was first patterned on a 300-nm-thick silicon dioxide (SiO_2_)-coated 4-inch silicon wafer using an image reversal photolithography process. A 200-nm-thick Au layer was then deposited onto the prepared PR mould substrate using an electron-beam (e-beam) evaporation technique. Prior to the Au deposition, a 10-nm-thick chromium (Cr) layer was deposited to promote adhesion between the substrate and the Au electrodes. Subsequently, the heating and probing electrodes were defined by selectively removing the unnecessary portion of the deposited Au using acetone in an ultrasonic bath at an operating frequency of 28 kHz. Then, a 100-nm-thick SiO_2_ film was deposited using a chemical vapour deposition (CVD) technique to electrically isolate the M13 virus from the heating electrode. The probing electrodes were exposed by chemically etching the SiO_2_ film through a photolithographically defined PR etching mask using a buffered oxide etchant (BOE) solution. The fabrication of the microheater chip was completed by dissolving the PR etching mask in an acetone bath at room temperature. Finally, virus colour pixels were fabricated by integrating the virus colour film onto the microheater chip through a well-optimised self-assembly process in a simple and reliable manner. Because the heating provides the driving force for the colour alteration, the heating performance of the fabricated microheater chip was examined (Supplementary Fig. 7a) before being integrated with the virus colour film. Supplementary Fig. 7b–d show the temperatures of the microheater chip in response to various applied voltages, indicating superior operational stability and reversibility of the device. Supplementary Fig. 8a schematically depicts our microheater-based colour-alteration system. Virus colour pixels were connected to the power supply by an aluminium wire. The power supply was automatically controlled by a home-built operating program. A charge-coupled device (CCD) video camera captured the colour alteration of the virus colour pixels, and a quantitative RGB colour-component analysis was performed on the captured images using a MATLAB program. Using the home-built operating device and the MATLAB analysis tool, we established a systemically controllable colour-generating device. As the voltage applied by the power supply was increased, successive alterations in colour were observed (Fig. 3b). The MATLAB RGB colour-component analysis facilitated delicate control of colour changes undistinguishable by the naked eye (Supplementary Fig. 8b). Even for an applied voltage of less than 1 V, the virus colour pixels showed significant colour alterations. This result demonstrates that virus colour pixels are promising candidates for use in low-power display devices. The colour alteration of virus colour pixels arises from external stimulation—in the present work, a heating effect (Supplementary Fig. S9). AFM measurements and an FFT analysis of AFM images were performed to demonstrate the role of heat in our system (Supplementary Fig. S10). Virus colour pixels were placed on the heating stage of the AFM, and structural changes were measured at varied temperature. When the FFT patterns are converted to spatial power spectrum, as shown in Supplementary Fig. 10, the spatial order of the virus colour pixels is clearly different throughout measurement. This is because the external heat vaporises the water inside the M13 phage bundles, leading to shrinkage of the bacteriophage bundle. The decreased bundle width and bundle-to-bundle distance lead to constructive interference of shorter wavelengths by coherent scattering of white light. When the external heat is removed, the M13 phage bundle absorbs water from the air and returns to its previous colour.

Upon removal of the external field, the M13 phage pixel colours were recovered without any noticeable change of optical properties (Fig. 4a). This shows that the colour pixels are durable and reversible even under repetitive operation (0 V to 1 V) (Supplementary Fig. S11). This desirable performance allows the virus pixels to be efficiently used as a display device platform. To demonstrate this, a 7-digit display panel integrated with seven individual microheaters, which are isolated electrically from one another, was fabricated by a single-step self-assembly process. With the application of an applied voltage of only 1 V, a clear colour alteration of the 7 segments was observed, as shown in Fig. 4b. Supplementary Fig. S12 also clearly shows that each segment of the 7-digit circuit can be operated individually and controlled selectively.

In summary, we developed an M13 phage-based temperature-dependent biomimetic colour-generation platform on a microheater chip. Through a combination of microheater technology and self-assembly of M13 phage, a new class of full colour display pixels was successfully demonstrated. The systematic colour alteration of M13 phage was easily achieved by controlling the temperature of the microheater chip in a precise and reversible manner. Our colour pixel platforms have multiple advantages over conventional colour display devices. First, we can fabricate natural colour pixels by using different kinds of substrates. We confirmed that the photonic crystal structures composed of M13 phage can generate a full range of RGB colours that can be sensitively switched using temperature changes controlled by a microheater chip. Second, given the simplicity of the fabrication process, the low voltage requirements and cycling stability, the virus colour pixels are a potential substitute for conventional colour pixels. The use of novel, biocompatible, highly uniform and chemically stable display designs based on virus colour pixels will be a promising approach for the development of various implantable, wearable and flexible devices in future.

## Methods

### Fabrication of M13 phage colour film

We prepared the virus colour film by utilizing simple pulling method. In this paper, the concentration is fixed. The Pulling speed and substrate materials were varied to generate desired colour. We used commercial syringe pump to control pulling speed precisely. (kd Scientific, Legato 180, Holliston, MA 01756, USA) Si wafer coated with ~5-nm-thick Pt adhesion layer and ~100-nm-thick Au film (Platypus technologies, 5520 Nobel Drive, Suite 100, Madison, WI 53711, USA), Si wafer with ~2-nm-thick native SiO_2_ layer (Uni nanotech, 119, Dongbaekjungang-ro, Giheung-gu, Yongin-si, Gyeonggi-do, Korea), Si wafer with Boron doped thermally grown ~300-nm-thick SiO_2_ layer (Uni nanotech, 119, Dongbaekjungang-ro, Giheung-gu, Yongin-si, Gyeonggi-do, Korea), glass slide(DURAN Group GmbH Otto-Schott-Str. 2197877 Wertheim/Main Germany) was utilized The 4E-type M13 phage that contenctration of 5 mg/ml in tris buffered saline (TBS) buffer (12.5 mM tris and 37.5 mM NaCl, pH 7.5) was used for preparing the colour film.

### Atomic Force Microscopy (AFM) measurement and Fast Fourier Transform (FFT) analysis of M13 phage color film

AFM images were collected using an NX10 AFM (Park Systems, Suwon, Korea) and operated by the data acquisition program XEP 3.0.4 (Park Systems, Suwon, Korea) and analysed by the image processing program XEI 1.8.2 (Park Systems, Suwon, Korea). All images were collected in true non-contact mode. Specialized probe for non-contact mode was selected for measurement, (PPP-NCHR, NANOSENSORS, Neuchatel, Switzerland) Temperature control stage was utilized for structural change measurement of virus colour film. (Park Systems, Suwon, Korea) Temperature control stage allows for precise control of temperatures ranging up to 250 °C with a 0.1 °C resolution. Fast Fourier Transform (FFT) analysis of AFM images was performed to confirm the periodicity of the virus based photonic crystal structure. Numerical computation of the Fourier transform was carried out in a 2D FFT algorithm in XEI 1.8.2.

### Reflectance measurement of M13 phage colour film

Virus colour film was illuminated by a white light which is generated by a Xenon lamp (X-Cite, Exfo, Mississauga, Canada). Reflected light was obtained using a fibre optic spectrophotometer (USB2000+, Ocean Optics, Dunedin, FL). Y-shaped reflection/backscattering optical fibre (QR400-7-VIS-NIR, Ocean Optics, Dunedin, FL) was used for both illumination and obtaining reflected light. An optical fibre fixed on a *z* stage was positioned normal to the virus colour film which had been fixed on a hollow rotation stage.

### Simulation of reflectance of virus colour film

Simulation processes were performed using commercial design and simulation tool Rsoft DiffractMOD™ (Synopsys, Inc., California, USA) which is based on the Rigorous Coupled Wave Analysis (RCWA) method. Numerical analysis of the AFM image was performed by utilizing the MATLAB program. (Mathworks Inc. Natick, MA) The raw AFM image was taken by 10 *μ*m × 10 *μ*m scale for MATLAB numerical analysis. Resulting height information was used for modelling upper layer of virus colour film. This layer cannot represent whole virus colour film because of limitation of the penetration depth of AFM tip. Underlying layer which had not been taken by AFM was simplified as a check shape layer of similar width with AFM image. Upper layer topology and thickness, underlying check layer topology and thickness and refractive index of substrate materials were chosen as a variables of simulation. Index resolution (x, y axis of 25 nm and z axis of 10 nm), refractive index of virus, scanning range (300 nm–900 nm), scanning step (25 nm) and direction of light (0°) was held.

## Additional Information

**How to cite this article**: Kim, W.-G. *et al.* Virus based Full Colour Pixels using a Microheater. *Sci. Rep.* **5**, 13757; doi: 10.1038/srep13757 (2015).

## Supplementary Material

Supplementary Information

## Figures and Tables

**Figure 1:**
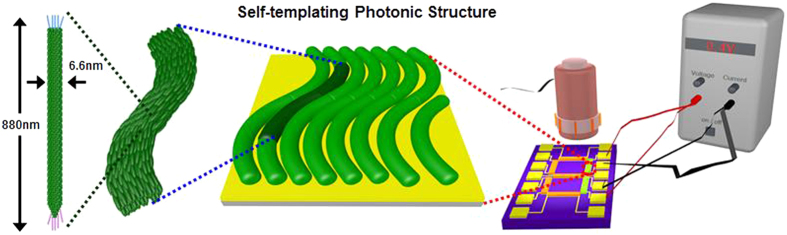
Diagram of the Bio-inspired colour-generation system. Inspired by the colour-generating photonic structure of mammalian skins and avian skin/feathers and their communication methods, we propose a novel full-colour display device for information delivery. In our system, self-assembly of M13 phage leads to a photonic crystal structure that serves as an RGB colour filter layer of a common display device. By adopting the virus colour film as a colour-generating source, there is minimal energy loss, in contrast to common colour displays.

**Figure 2:**
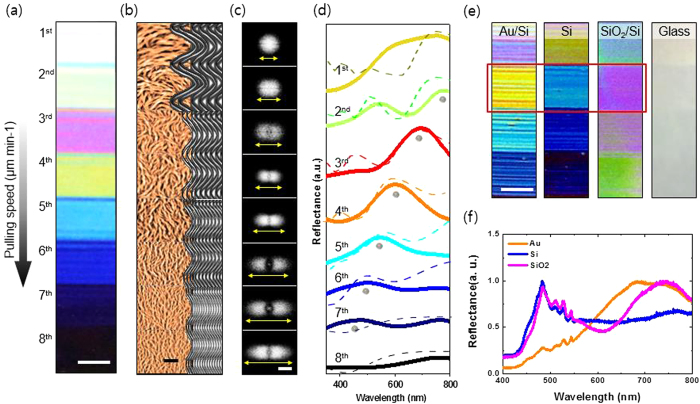
Modification of M13 phage colour film by various processes. (**a**) With a simple pulling method, an M13 phage photonic crystal structure was deposited on an Au wafer. We fabricated various different colour bands by precisely modulating the pulling speed. Scale bar = 1 cm (**b**) AFM image set of a self-assembled M13 phage nanostructure (left, AFM image; right, schematic diagrams). Measurements were taken at each band. With increasing pulling speed, the bundle width and bundle-to-bundle distance decrease. As a result of the dense nanostructure, the scattering wavelength shifts from red to blue. Scale bar = 5μm (**c**) Fast Fourier transform (FFT) analysis of the AFM image was performed to demonstrate the quantitative difference of the spatial order of the colour bands. A denser photonic crystal nanostructure leads to a more diffuse FFT pattern. Scale bar = 1.6 μm^−1^ (**d**) Reflectance spectrum (full line) and simulated reflectance spectrum (dashed line) of the virus colour film. All spectra were normalized to demonstrate the tendency of peak wavelengths. (**e**) Virus colour films deposited on different substrates. Each colour film was assembled under the same conditions. They showed different colours despite having photonic crystal structures of the same spatial order. Scale bar = 1 cm (**f**) Reflectance measurement confirmed the effect of the substrate materials.

**Figure 3:**
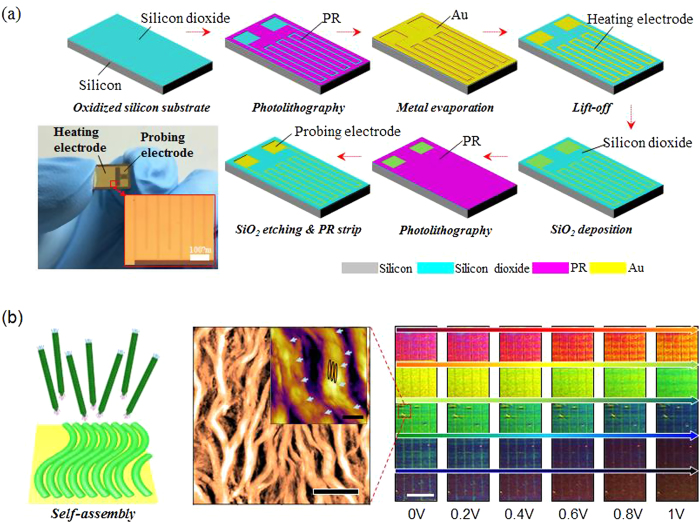
Fabrication of a microheater chip and operation of the virus colour pixels with a microheater. (**a**) The temperature-controllable microheater chip was fabricated by a standard metal lift-off process (**b**) Virus colour pixels were deposited onto a microheater, which was fabricated using simple photolithography. An applied voltage caused a significant alteration of the colour. Scale bars = 5 μm (inset = 1μm), and 1 cm, respectively.

**Figure 4:**
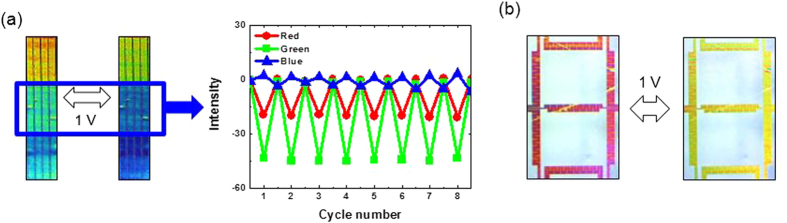
Durability of a virus colour film and an advanced display panel. (**a**) Reversibility and durability tests of virus colour pixels using a MATLAB RGB colour component analysis program. 1 V was periodically applied to the microheater while the MATLAB program analysed images captured by a CCD camera. (**b**) A 7-segment panel was fabricated by a metal lift-off process. A simple pulling method leads to self-assembly of M13 phage on the 7-segment panel. A voltage of ~1 V was applied to the 7-segment display to operate the virus colour pixels.

## References

[b1] Bowler P. J. Evolution: The History of an idea. University of California Press, Oakland, CA (1984).

[b2] Morris S. C. Evolution: Bringing molecules into the fold. Cell 100, 1–11 (2000). 10647927 10.1016/s0092-8674(00)81679-7

[b3] Crookes W. J. *et al.* Reflectins: The Unusual Proteins of Squid Reflective Tissues. Science 303, 235–238 (2004). 14716016 10.1126/science.1091288

[b4] Sharma V., Crne M., Park J. O. & Srinivasarao M. Structural Origin of Circularly Polarized Iridescence in Jeweled Beetles. Science 325, 449–451 (2009). 19628862 10.1126/science.1172051

[b5] Gao X. & Jiang L. Water-repellent legs of water striders. Nature 432, 36 (2004). 15525973 10.1038/432036a

[b6] Aizenberg J., Tkachenko A., Weiner S., Addadi L. & Hendler G. Calcitic microlenses as part of the photoreceptor system in brittlestars. Nature 412, 819–822 (2001). 11518966 10.1038/35090573

[b7] Prum R. O., Quinn T. & Torres R. H. Anatomically diverse butterfly scales all produce structural colours by coherent scattering. J. Exp. Biol. 209, 748–765 (2005). 16449568 10.1242/jeb.02051

[b8] Bradbury J. W. & Vehrencamp S. L. Principles of animal communication. Sinauer Associates, Sunderland, MA (1998).

[b9] Prum R. O. & Torres R. Structural colouration of avian skin: convergent evolution of coherently scattering dermal collagen arrays. J. Exp. Biol. 206, 2409–2429 (2003). 12796458 10.1242/jeb.00431

[b10] Prum R. O. & Torres R. Structural colouration of mammalian skin: convergent evolution of coherently scattering dermal collagen arrays. J. Exp. Biol. 207, 2157–2172 (2004). 15143148 10.1242/jeb.00989

[b11] Arsenault A. C., Puzzo D. P., Manners I. & Ozin G. A. Photonic-crystal full-colour display. Nature photon. 1, 468–472 (2007).

[b12] Cai. Z. *et al.* 2D Photonic crystal protein hydrogel coulometer for sensing serum albumin ligand binding. Anal. Chem. 86, 4840–4847 (2014). 24766373 10.1021/ac404134t

[b13] Zhang J. T. *et al.* 2-D Array photonic crystal sensing motif. J. Am. Chem. Soc. 133, 9152–9155 (2011). 21604702 10.1021/ja201015c

[b14] Muscatello M. M. W. & Ahser S. A. Poly (vinyl alcohol) rehydratable photonic crystal sensor materials. Adv. Funct. Mater. 18, 1–8 (2008). 10.1002/adfm.200701210 PMC3111221 21666875

[b15] Oh J. W. *et al.* Biomimetic virus-based colourimetric sensors. Nature commun. 5, 3043 (2014). 24448217 10.1038/ncomms4043

[b16] Lee H., Lee B. P. & Messersmith P. B. A reversible wet/dry adhesive inspired by mussels and geckos. Nature 448, 338–342 (2007). 17637666 10.1038/nature05968

[b17] He B., Patankar N. A. & Lee J. Multiple equilibrium droplet shapes and design criterion for rough hydrophobic surfaces. Langmuir 19, 4999–5003 (2003).

[b18] Geim A. K. *et al.* Microfabricated adhesive mimicking gecko foot-hair. Nature Mater. 2, 461–463 (2003). 12776092 10.1038/nmat917

[b19] Yang S. *et al.* Functional Biomimetic Microlens Arrays with Integrated Pores. Adv. Mater. 17, 435–438 (2005).

[b20] Walish J. J., Kang Y., Mickiewicz R. A. & Thomas E. L. Bioinspired Electrochemically Tunable Block Copolymer Full Color Pixels. Adv. Mater. 21, 3078–3081 (2009).

[b21] Lim H. S., Lee J. H., Walish J. J. & Thomas E. L. Dynamic Swelling of Tumable Full-Color Block Copolymer Photonic Gels via Countgerion Exchange. ACS Nano 6, 8933–8939 (2012). 23020142 10.1021/nn302949n

[b22] Kolle M. *et al.* Mimicking the colourful wing scale structure of the Papilio blumei butterfly. Nature Nanotech. 5, 511–515 (2010). 10.1038/nnano.2010.101 20512131

[b23] Suzuki K., Hamachi Y. & Baba T. Fabrication and characterization of chalcogenide glass photonic crystal waveguides. Opt, Express 17, 22393–22400 (2009). 20052163 10.1364/OE.17.022393

[b24] Whitesides G. M. & Grzybowski B. Self-Assembly at All Scales. Science 295, 2418–2421 (2002). 11923529 10.1126/science.1070821

[b25] Xia Y., Yin Y., Lu Y. & McLellan J. Template-assisted self-assmbly of spherical colloids into complex and controllable structures. Adv. Funct. Mater. 12, 907–918 (2003).

[b26] He L. *et al.* Self-assembly and magnetically induced phase transition of three-dimensional colloidal photonic crystals. Nanoscale 4, 4438–4442 (2012). 22692448 10.1039/c2nr31068f

[b27] Nikoobakht B., Wang Z. L. & El-sayed M. A. Self-assembly of gold nanorods. J. Phys. Chem. B. 104, 8635–8640 (2000).

[b28] Sau T. K. & Murphy C. J. Seeded high yield synthesis of short Au nanorods in aqueous solution. Langmuir 20, 6414–6420 (2004). 15248731 10.1021/la049463z

[b29] He Y. *et al.* Hierarchical self-assembly of DNA into symmetric supramolecular polyhedra. Nature 452, 198–202 (2008). 18337818 10.1038/nature06597

[b30] He J. *et al.* Self-assembly of tobacco mosaic virus at oil/water interfaces, Lanmuir 25, 4979–4987 (2009). 10.1021/la803533n 19397351

[b31] Cisneros D. A., Hung C., Franz C. M. & Muller D. J. Observing growth steps of collagen self-assembly by time-lapse high-resolution atomic force microscopy. J. Struct. Biol. 154, 232–245 (2006). 16600632 10.1016/j.jsb.2006.02.006

[b32] Lee Y. J. *et al.* Fabricating genetically engineereed high-power lithium-ion batteries using multiple virus genes. Sience 324, 1051–1055 (2009). 10.1126/science.1171541 19342549

[b33] Courchesne N. M. D. *et al.* Assembly of a bacteriophage-based template for the organization of materials into nanoporous networks. Adv. Mater. 26, 3398–3404 (2014). 24648015 10.1002/adma.201305928 PMC4043913

[b34] Yoo P. J., Nam K. T., Belcher A. M. & Hammond P. T. Solvent-Assisted Patterning of polyelectrolyte Multilayers and selective deposition of virus assemblies. Nano Letters 8, 1081–1089 (2008). 18355056 10.1021/nl073079f

[b35] Chung W. J. *et al.* Biomimetic self-templating supramolecular structures. Nature 478, 364–368 (2011). 22012394 10.1038/nature10513

[b36] Lee B. Y. *et al.* Virus - based piezoelectric energy generation. Nature nanotech. 7, 351–356 (2012). 10.1038/nnano.2012.69 22581406

[b37] Doucet S. M., Shawkey M. D., Hill G. E. & Montgomerie R. Iridescent plumage in satin bowerbirds: structure, mechanisms and nanostructural predictors of individual variation in colour. J. Exp. Biol. 209, 380–390 (2006). 16391360 10.1242/jeb.01988

