# Supplementary material for: Virus based Full Colour Pixels using a Microheater
Source: Sci Rep. 2015 Sep 3;5:13757. doi: 10.1038/srep13757 (PMC4558721; doi:10.1038/srep13757)
Supplement: Supplementary Information [file srep13757-s1.doc]

**Supplementary Information**

**Virus based Full Colour Pixels using a Microheater**

*Won-Geun Kim^1,†^, Kyujung Kim^2, †^, Sung-Hun Ha^3^, Hyerin Song^2^, Hyun-Woo Yu^3^, Chuntae Kim^3^, Jong-Man Kim^3^,* and Jin-Woo Oh^1,3^,**

*^1^Department of Nanomaterials Engineering, ^2^Department of Cogno-Mechatronics Engineering,*

*^3^Department of Nano Fusion Technology, Pusan National University, Busan 609-735, Republic of Korea*

***†****These authors contributed equally to this work.*

**Corresponding author E-mail: ojw@pusan.ac.kr (J.-W. Oh) &* [*jongkim@pusan.ac.kr*](mailto:jongkim@pusan.ac.kr) *(J.-M. Kim)*

**Methods and Materials**


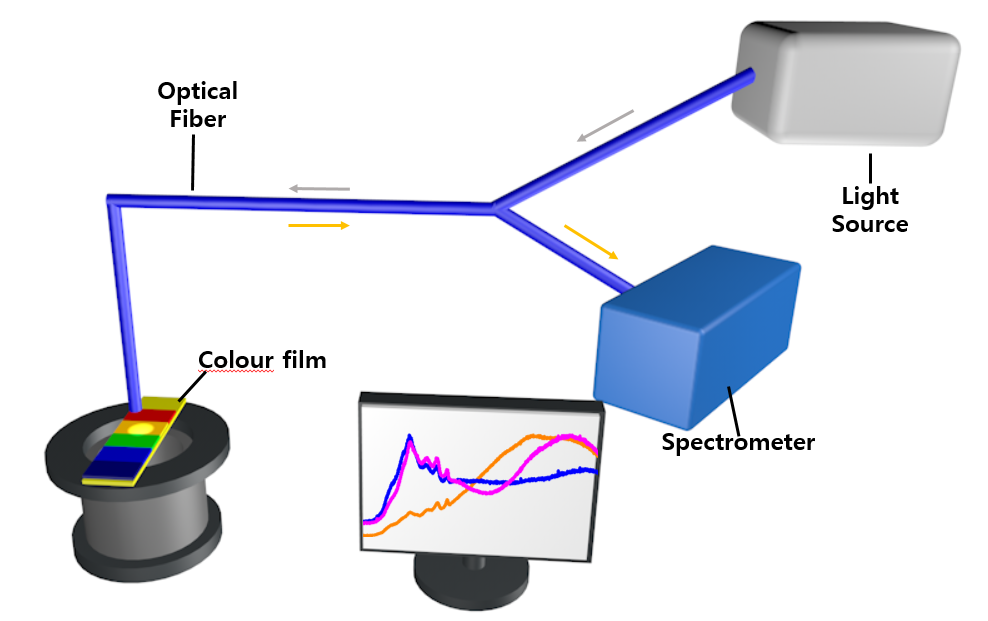


**Reflectance measurement of M13 phage colour film.** Virus colour film was illuminated by a white light which is generated by a Xenon lamp (X-Cite, Exfo, Mississauga, Canada). Reflected light was obtained using a fibre optic spectrophotometer (USB2000+, Ocean Optics, Dunedin, FL). Y-shaped reflection/backscattering optical fibre (QR400-7-VIS-NIR , Ocean Optics, Dunedin, FL) was used for both illumination and obtaining reflected light. An optical fibre fixed on a *z* stage was positioned normal to the virus colour film which had been fixed on a hollow rotation stage.

**Home-built program for automatic operation of power supply.**

Control I/O of Agilent E3649A power supply using .NET framework

1. Agilent E3649A controller abstract

a. We developed a .NET based controller with cheap RS-232 interface. By using this application to achieve a good controllability over the Agilent E3649A power supply. We manipulated the voltage input signal and gained the current and resistance output precisely for a long period, ultimately making it possible to synchronize the voltage input and temperature out which was obtained from the other measurement (*Testo 845*) successfully based on the time data as a parameter.

b. To get a precise set of the input and out data with RS-232 interface, instead of conventional for loop which is implanted serially and widely used in laboratories, we adopted a dual thread parallel programming by which, time-measurement and I/O measurement were done in parallel leading to a precise measurement.

1. Code explanation

a. Method 1

i. The application attempts to connect to the device via Agilent E36XX IVI driver. All the commands are using SCPI (Standard Commands for Programmable Instruments) to control the I/O of the power supply.

b. Method 2

i. The measurement starts with implementing the method “btn_measure_Click”. The variable “str_builder” is global variable and reset every start of another measurement. Instead of storing the measurement data at the end of the measurement, the application stores the data each time to prevent unwanted loss of the data by using “StringBuilder” class which can write data on the file in real time during measurement.

ii. The method “System.Threading.Timer” invokes the method “Measurement” which inputs the signals to and receives data from the device with a predefined measurement period “numericUpDown_interval.Text” variable until it is stopped by application. Instead of using for loop statement, by taking advantage of the thread invoking, it is possible to control and get a data from the device with a precise time scale.

c. Method 3

i. The method “Measurement” is invoked continuously by the “Method 2” with the predefined interval.

ii. The method gets actual voltage, current and resistance data from and gives a command to the device as a form of voltage value indirectly.

iii. The received data are stored in a form of “csv” file with a current measurement time.

iv. The application also has a sweep function which changes the displayed voltage value of the “NumericUpDown” instance. When the value of this instance is changed, the method “numericUpDown_voltage_ValueChanged” is implemented. This invokes the method “ApplyVoltage” which attempts to change the voltage actually and directly.

1. Digest of the actual code

a. Redundant lines which are not necessary for the explanation were removed. The code in this table will not be implemented without remaining lines.

| # | Method digest |
| --- | --- |
| 1 | public void ConnectToDevice(string str_deviceName)  {  try  {  e3649a.Initialize(str_deviceName, false, true);  e3649a.WaitForOperationComplete(500);  e3649a.Status.Clear();  }  catch  {  //Scenario for a connection failure.  } |
| 2 | private void btn_measure_Click(object sender, EventArgs e)  {  try  {  str_builder = new StringBuilder();  str_builder.AppendLine("T,T(ms) - Measure first,V(V),I(A),R(Ohm)");    timer_measure = new System.Threading.Timer(new TimerCallback(Measurement), null, 100, Int32.Parse(numericUpDown_interval.Text));  stopwatch.Start();  }  catch (Exception ex)  {  //Scenario for a measurement failure.  }  } |
| 3 | private void Measurement(object obj)  {  Main_copy.Invoke(new MethodInvoker(delegate()  {  try  {  dt_time = DateTime.Now;  string str_now = dt_time.Month.ToString("D2") + dt_time.Day.ToString("D2") + "-" + dt_time.Hour.ToString("D2") + ":" + dt_time.Minute.ToString("D2") + ":" + dt_time.Second.ToString("D2");  double dbl_voltage = e3649a.Outputs.get_Item(str_output).Measure(AgilentE36xxMeasurementTypeEnum.AgilentE36xxMeasurementVoltage);  double dbl_current = e3649a.Outputs.get_Item(str_output).Measure(AgilentE36xxMeasurementTypeEnum.AgilentE36xxMeasurementCurrent);  double dbl_resist = dbl_voltage / dbl_current;  tb_measure_voltage.Text = dbl_voltage.ToString();  tb_measure_current.Text = dbl_current.ToString();  tb_measure_resistance.Text = dbl_resist.ToString();  if (bool_measure)  {  str_builder.AppendLine(str_now + "," + stopwatch.ElapsedMilliseconds.ToString() + "," + dbl_voltage.ToString() + "," + dbl_current.ToString() + "," + dbl_resist.ToString());  }  if (chk_sweep.Checked)  {  if(dbl_sweepitem.Length != int_measureCount)  {  numericUpDown_voltage.Value = (decimal)dbl_sweepitem[int_measureCount];  listBox_sweep.SetSelected(int_measureCount, true);  int_measureCount++;  }  else  {  btn_stop.PerformClick();  group_current.Enabled = false;  }  }  }  catch (Exception)  {  //Scenario for a measurement failure  }  }));  } |
| 4 | private void numericUpDown_voltage_ValueChanged(object sender, EventArgs e)  {  Main_copy.ApplyVoltage(this.int_channel, (double)numericUpDown_voltage.Value);  } |
| 5 | public void ApplyVoltage(int int_channel, double dbl_voltage)  {  string str_output=SelectChannel(int_channel,1);  string str_channel=SelectChannel(int_channel,2);  try  {  e3649a.get_Item(str_output).VoltageLevel = dbl_voltage;  e3649a.Outputs.Enabled = true;  }  catch (Exception)  {  //Scenario for a voltage control failure  }  } |


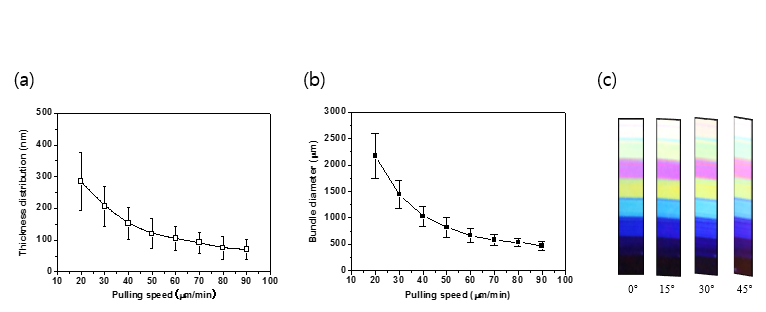


**Supplementary Figure 1. Thickness of the virus colour film for simulation and view angle independent colour of virus colour film under omni-directional illumination.** The virus colour film shows various nanostructures due to the pulling speed. **a,** As increasing the pulling speed, the thickness of the virus colour film becomes decreasing. This is because of a deficiency of time for self-assembly of a M13 phage. **b,** By the same token, the diameter of M13 phage bundle also decreasing due to the increasing pulling speed. **c,** The virus colour film shows a little colour alteration even if viewing angle is tuned. We took the images of the same virus colour film while viewing angle was tuned. There are almost no colour alteration between viewing angle of 0° to 45° with a 15° step.


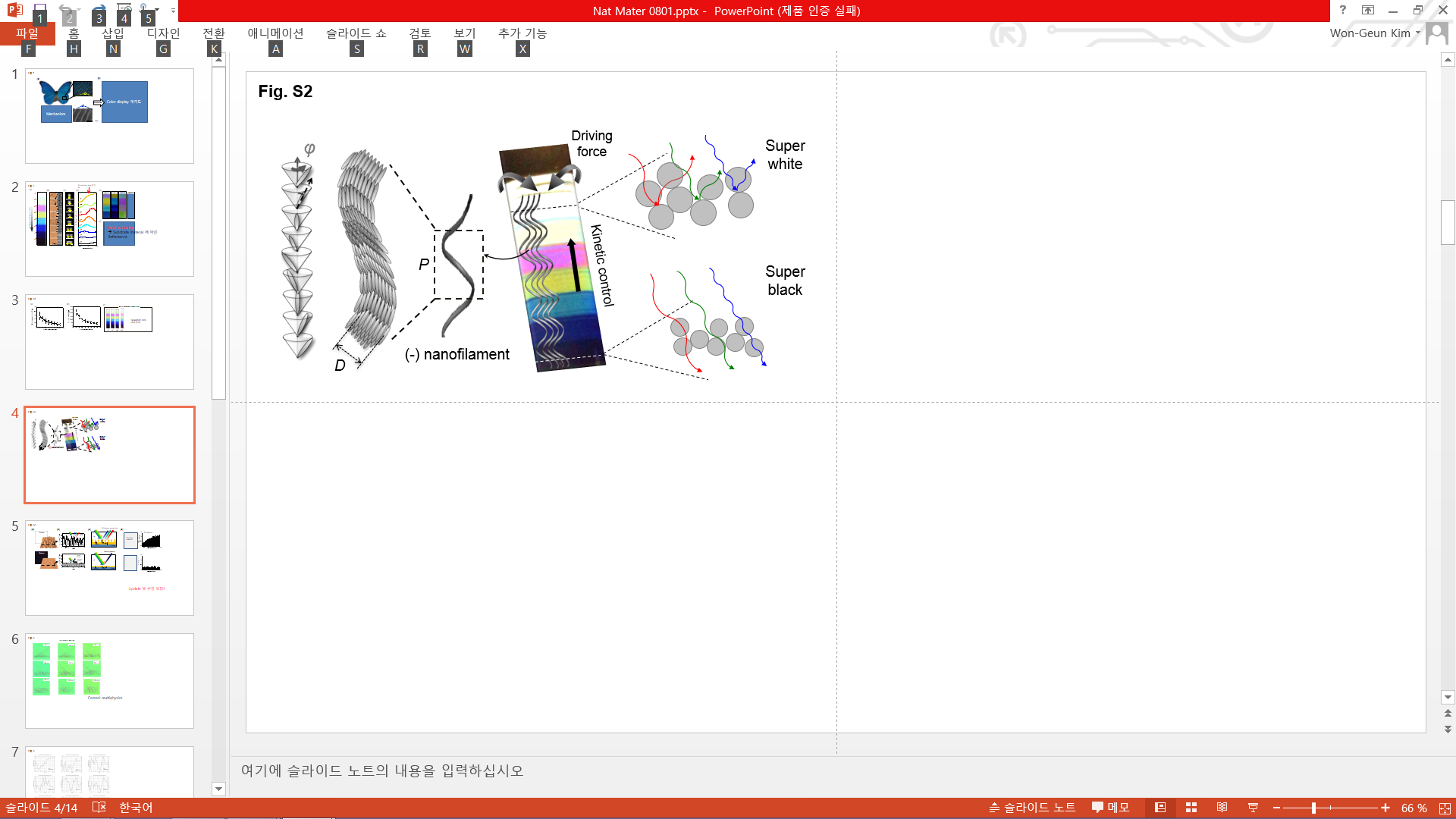


**Supplementary Figure 2. Schematic illustration of the formation of virus colour film structure.** Simple pulling process leads to a self-assembly of the M13 phage. At the edge of the substrate, there are relatively large surface area compared with the centre of substrate. As a result, an evaporation of a solvent is dominant in the edge and therefore the M13 phage is more accumulated in the edge than the centre. Concentration gradient between the edge and the centre leads to diffusion of M13 phage. In addition to the liquid crystal phase transition, edge-to-centre diffusion is also the driving force of smectic helicoidal nanofilament(SHN) structure formation. By controlling pulling speed, we can generate various spatial order of SHN.


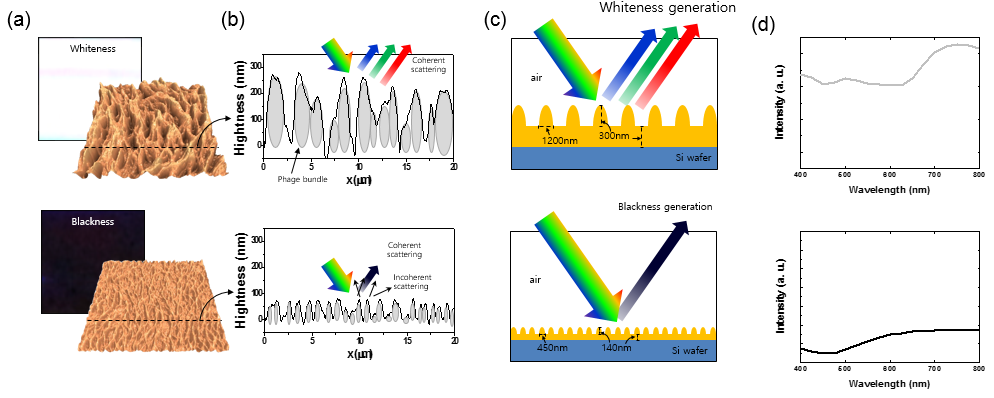


Supplementary Figure 3. Schematic illustration of the super white and super black generation Mechanism. a, The AFM image of the whiteness generating structure and the blackness generating structure b, Cross sectional profiles of the corresponding AFM images c, Schematic illustration of each structure and whiteness/blackness generation. d, Calculated reflectance spectra obtained from the AFM images.


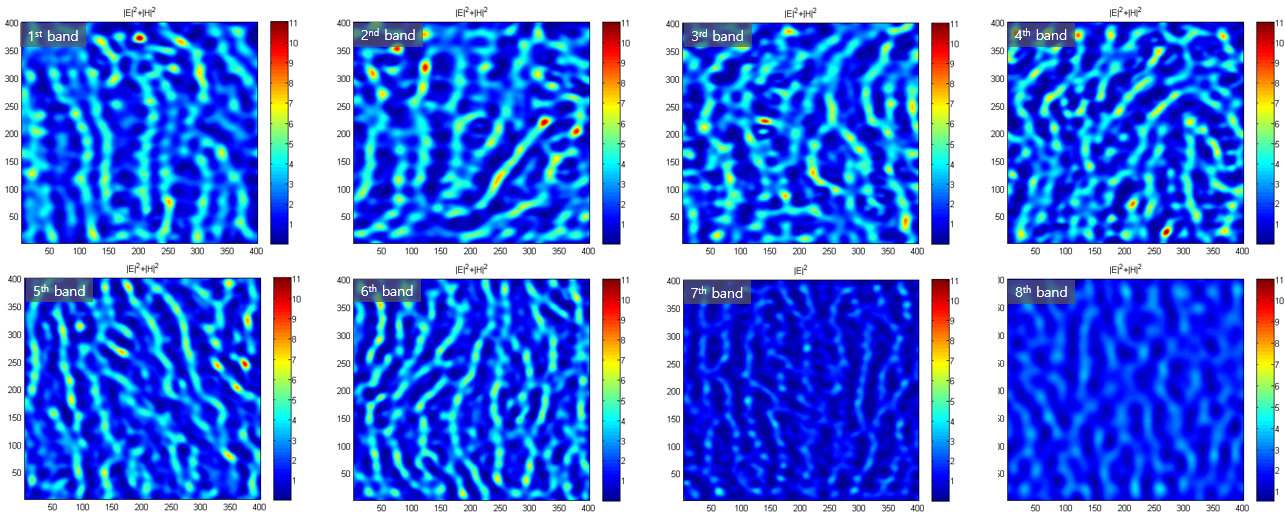


**Supplementary Figure 4. Maximum near field intensity distributions of each band at the specific wavelengths.** Different conformations of bands are presenting wavelength-dependent near field distributions. Each near field was calculated at 750 nm (1st), 750 nm (2nd), 700 nm (3rd), 600 nm (4th), 525 nm (5th), 500 nm (6th), 425 nm (7th), 700 nm (8th) orderly.


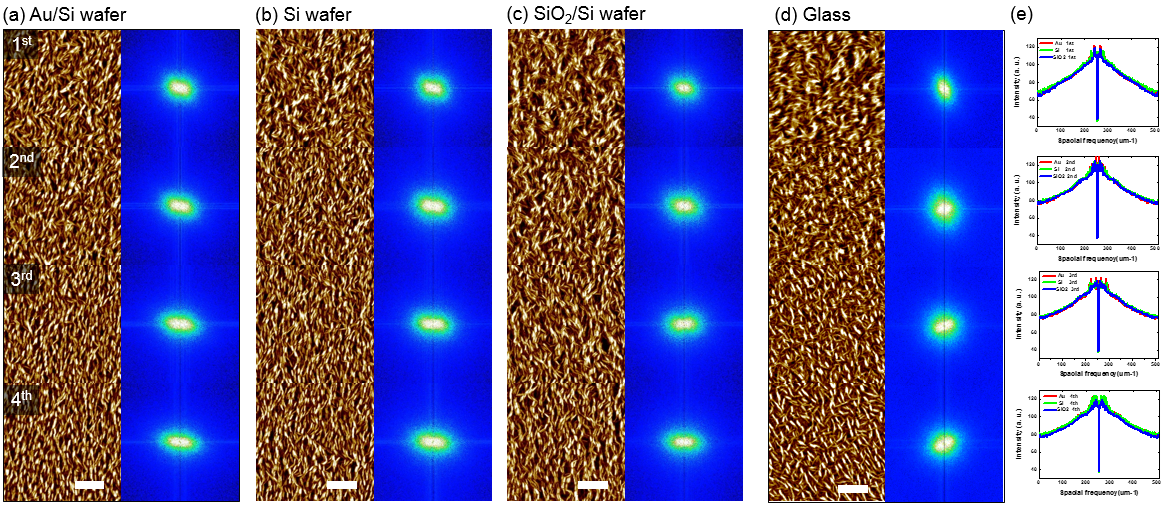


**Supplementary Figure 5. FFT analysis of AFM images of M13 bacteriophage colour film on various substrate.** AFM images of the virus colour films which were fabricated on **a,** 100-nm-thick Au on Si wafer, **b,** native SiO_2_ on Si wafer, **c,** thermally grown 200-nm-thick SiO_2_ on Si wafer, and **d,** glass slide(scale bar = 5μm) and FFT analysis of each AFM images. **e,** According to FFT analysis, virus colour films which were fabricated in the same conditon had an equivalent spatial order .


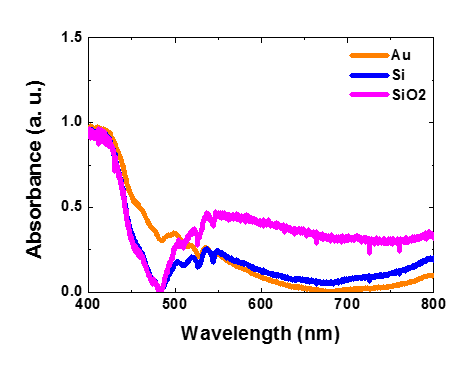


**Supplementary Figure 6. Absorbance spectrum of the back-substrates**. Each substrate has the different optical properties depend on its chemical component.


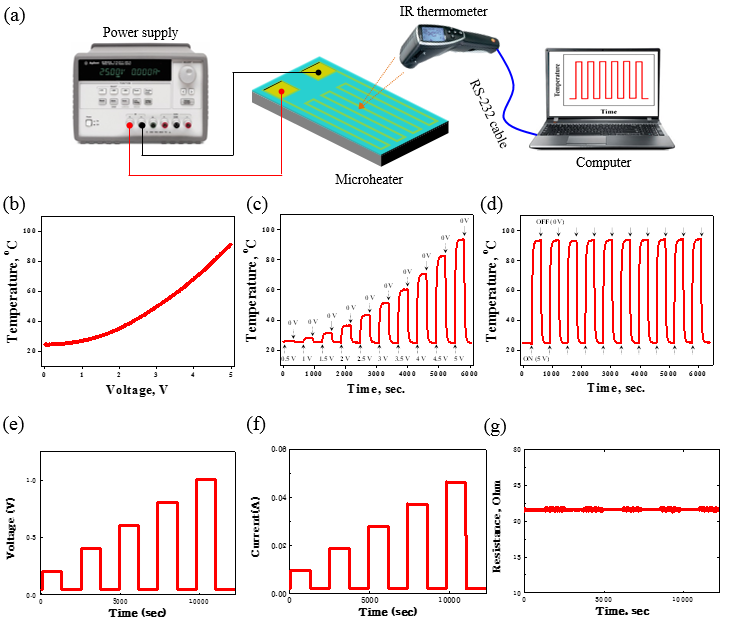


**Supplementary Figure 7. Characterization of microheater Chip.** **a,** Experimental setup for electro-thermal characterization of microheater. The heating performance of the fabricated microheater was characterized by observing change in temperature with applied voltage after electrically wiring using a silver paste. A DC voltage was applied to the microheater using a power supply (E3631A, Agilent Technologies). The resulting temperature of the microheater was measured and recorded in real time using an infrared (IR) thermometer (Model 845, Testo) connected with a computer through a RS-232 data cable. **b,** Temperature changes of the microheater as a function of applied voltage. The temperature of the microheater chip with an initial resistance of ~27 Ω was exponentially increased with increasing the applied voltage, representing the maximum temperature of ~91 °C at a voltage of 5 V. **c,** Temperature changes of the microheater with respect to applied voltage for up to 5 V with a step of 0.5 V. The results clearly show that the microheater can be digitally operated in response to the stepped voltage inputs. Moreover, the steady-state temperatures of the chip at each excited stage were also exponentially increased with increasing the applied voltage, agreeing well with the results of (b). This suggests that the proposed microheater chip can be an efficient driving force for digital colour alteration of M13 virus pixel. **d,** Temperature changes of the microheater under repetitive application of voltage (0 V to 5 V) for up to 10 cycles. The microheater stably responded with the cyclic voltage inputs, while representing uniform heating performance with a minimal standard deviation of ~0.2 °C at the steady-states for the 10 cycles. This suggests that M13 virus device can be employed as a new class of display pixel after integrating with a microheater chip thanks to the reliable operations of the microheater platform.


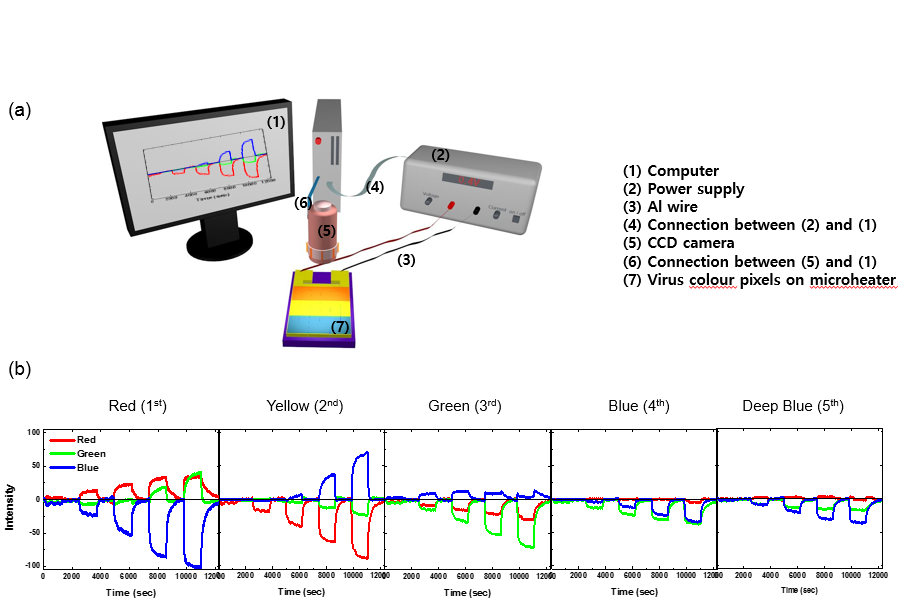


**Supplementary Figure 8.** **Automatic operation-measurement system.** We established an automatic system for a precise and convenient operation and measurement of the virus colour pixels. **a**, Schematic illustration of the automatic operation-measurement system. The microheater was connected to the power supply through the aluminium wire. The CCD camera captured images during operation in real time. The power supply was connected to the computer system for an automatic operation. The CCD camera also connected to the computer system and the MATLAB colour component analysis program performed analysis of images. **b**, The results of MATLAB colour component analysis. When a voltage of 0.2 V was applied to the microheater chip, a colour change is undistinguishable with naked human eyes.


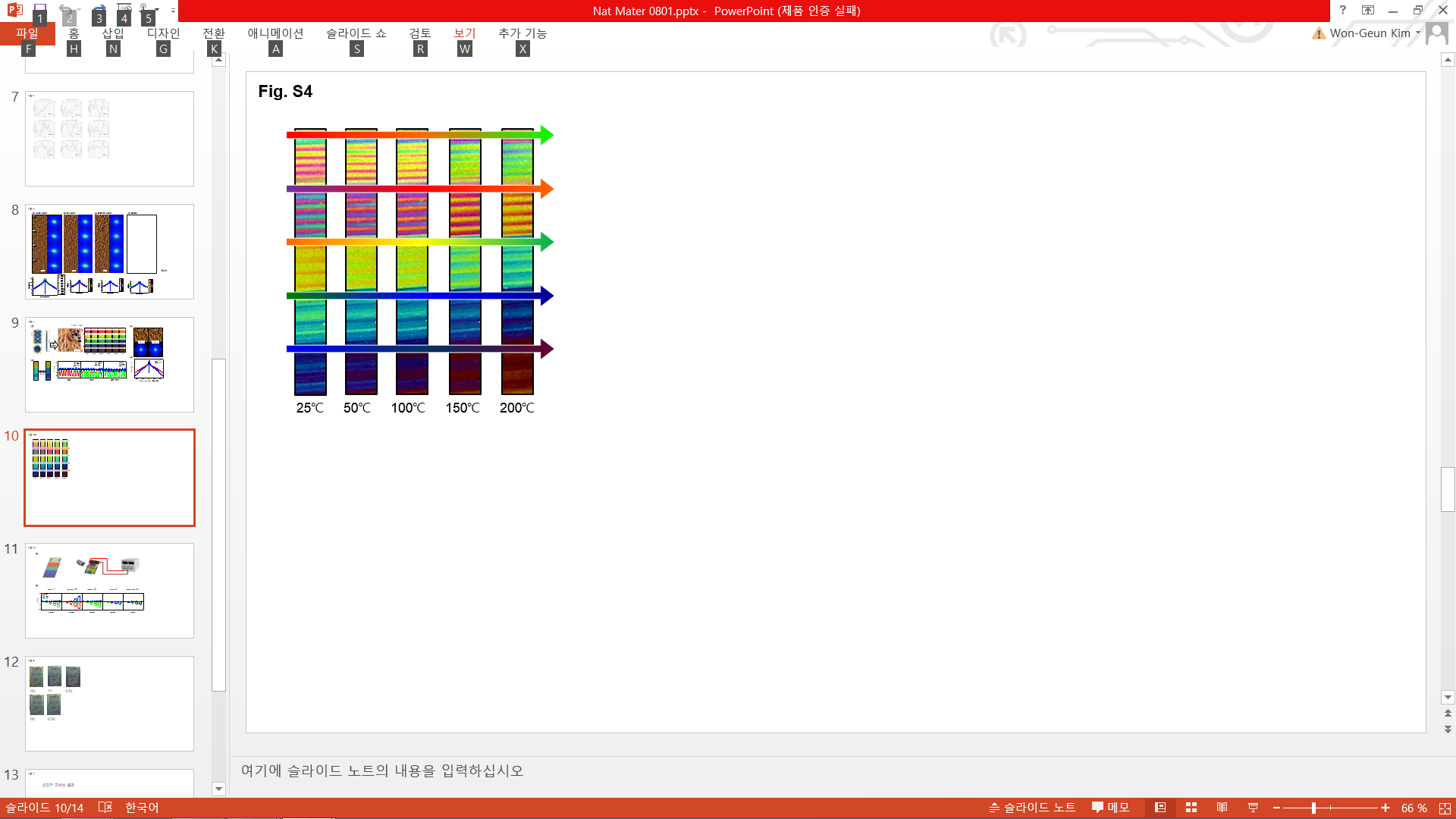


**Supplementary Figure 9. Colour alteration of the virus colour pixels by applied heat.** Temperature is the driving force of a colour alteration of the virus colour pixels. We captured the images of a sample while continuously changing the temperature.


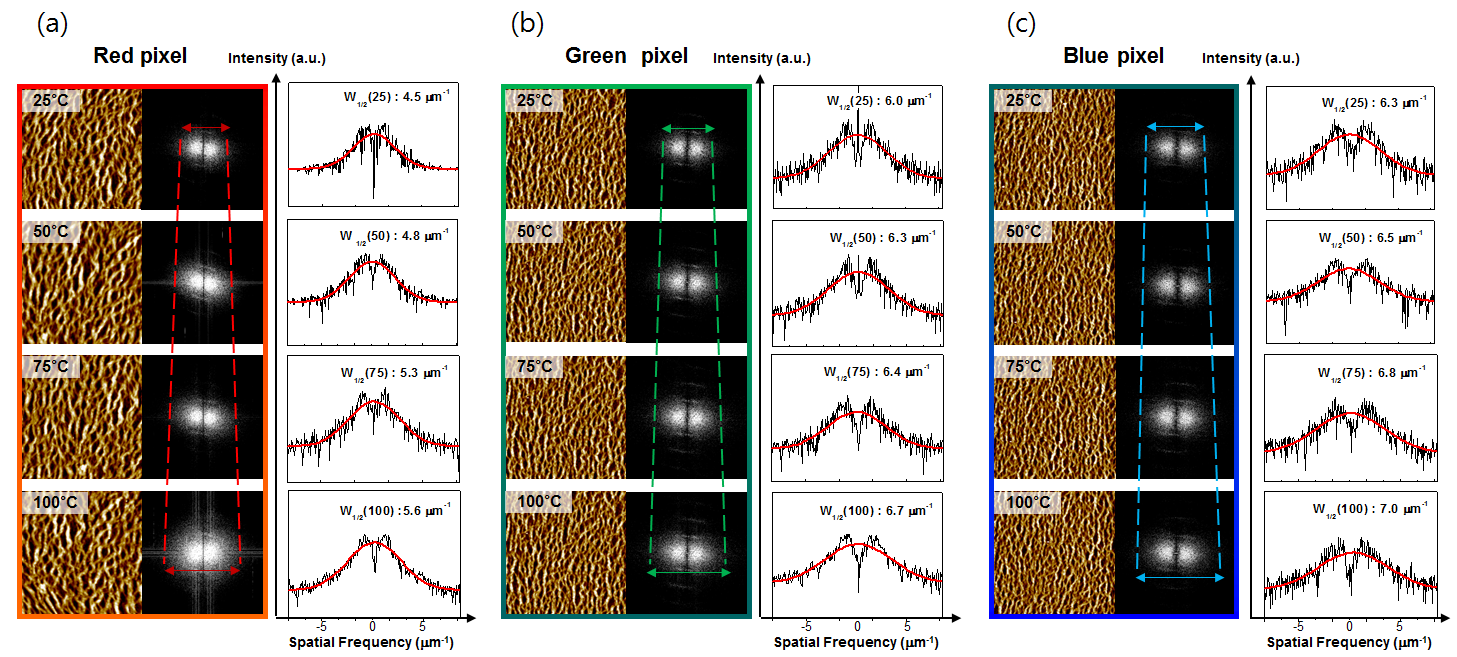


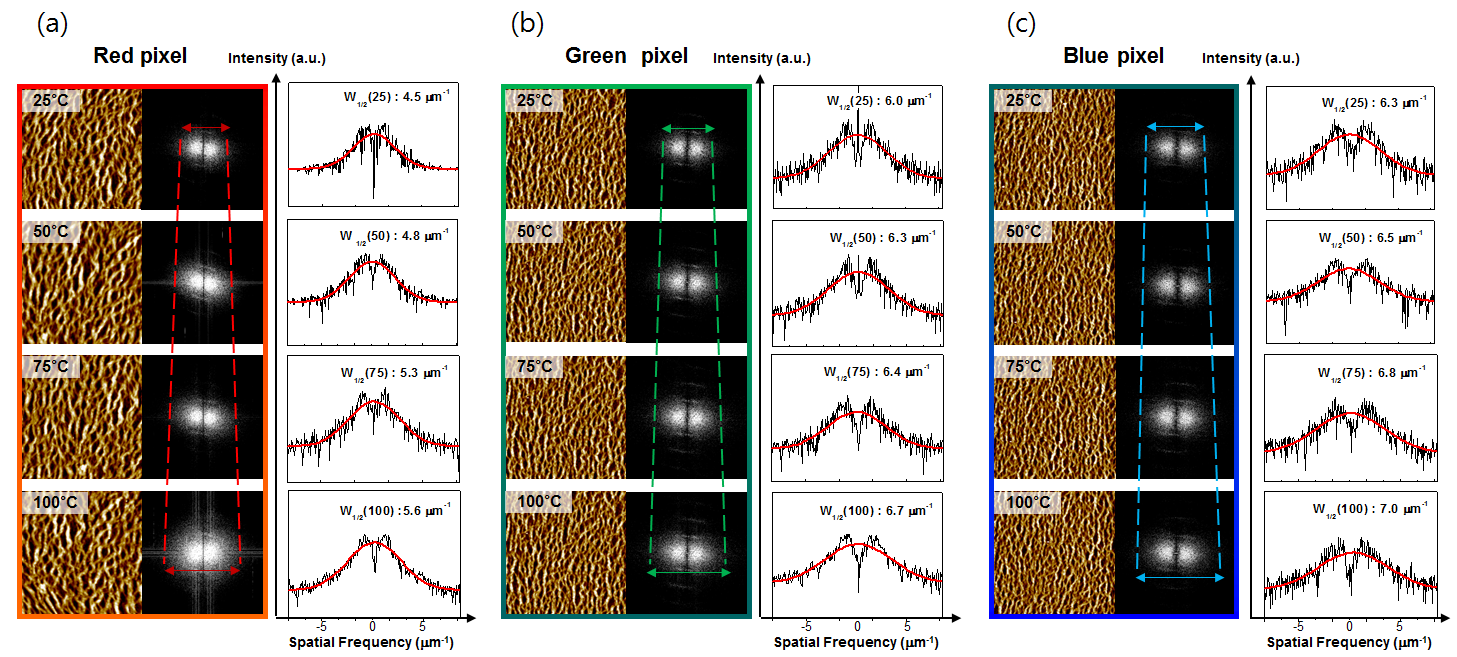


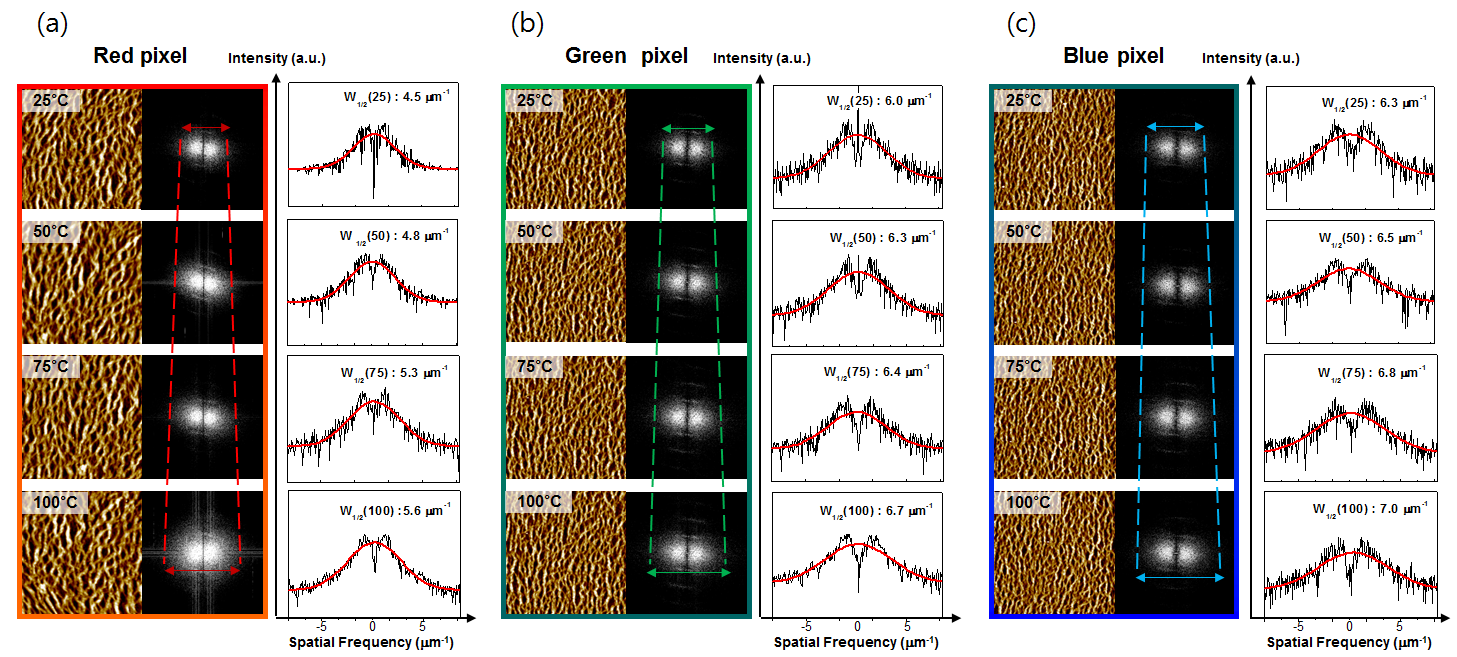


**Supplementary Figure 10. AFM images and two-dimensional fast Fourier transformation(FFT) analysis to investigate the colour alterating mechanism of virus nanostructure.** AFM images and two-dimensional FFT images of **a,** Red pixel, **b,** Green pixel and **c,** Blue pixel at varied temperature. Each pixel is composed of different diameter virus bundle by controlling the pulling speed. A different periodicity of phage bundles within each pixel can exhibit a different colour. For more quantitative analysis, comparison of the spatial power spectra of each pixel was performed. When temperature is increased, the width of spatial power spectrum was widened due to the increased high frequency components. This is because of more narrowly spaced virus bundles through shrinkage. The fitting curve was calculated by Gaussian function (w1/2: full width at half maximum).


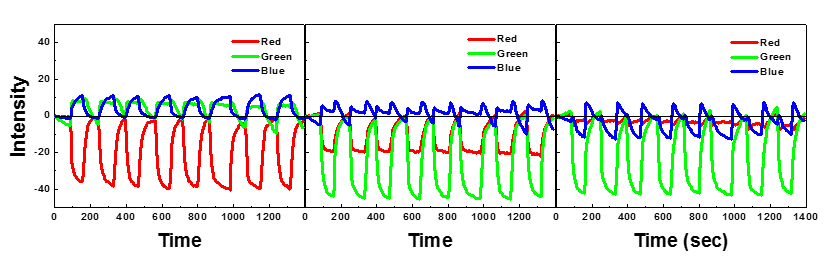


**Supplementary Figure 11. MATLAB RGB colour component analysis for the durability test of the virus colour film.** MATLAB RGB colour component analysis program analysed the images which were captured during the durability test.


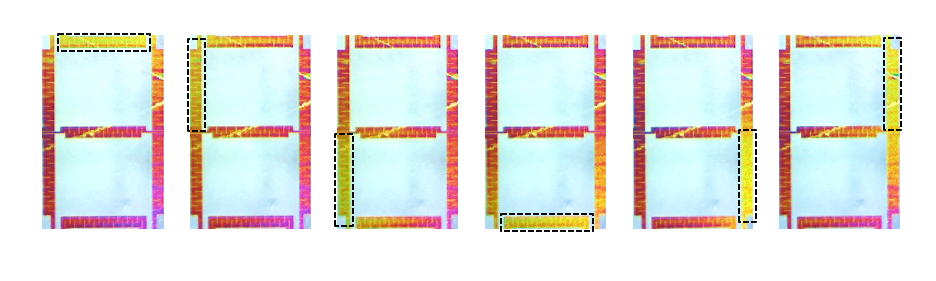


**Supplementary Figure 12. Controlled operation of each segment on the 7-digit pannel.** 7-digit panel was also fabricated by a standard metal lift-off process. Dot boxes indicate segment activated with applied voltage of 1 V.
